# Investigation of Radiological and Chemical Contents of Bauxite Ore Extracted in Turkey

**DOI:** 10.1021/acsomega.2c04203

**Published:** 2022-10-25

**Authors:** Aydan Altıkulaç

**Affiliations:** Ula Ali Koçman Vocational School, Muǧla Sıtkı Koçman University, 48640 Ula, Muǧla 48000, Turkey

## Abstract

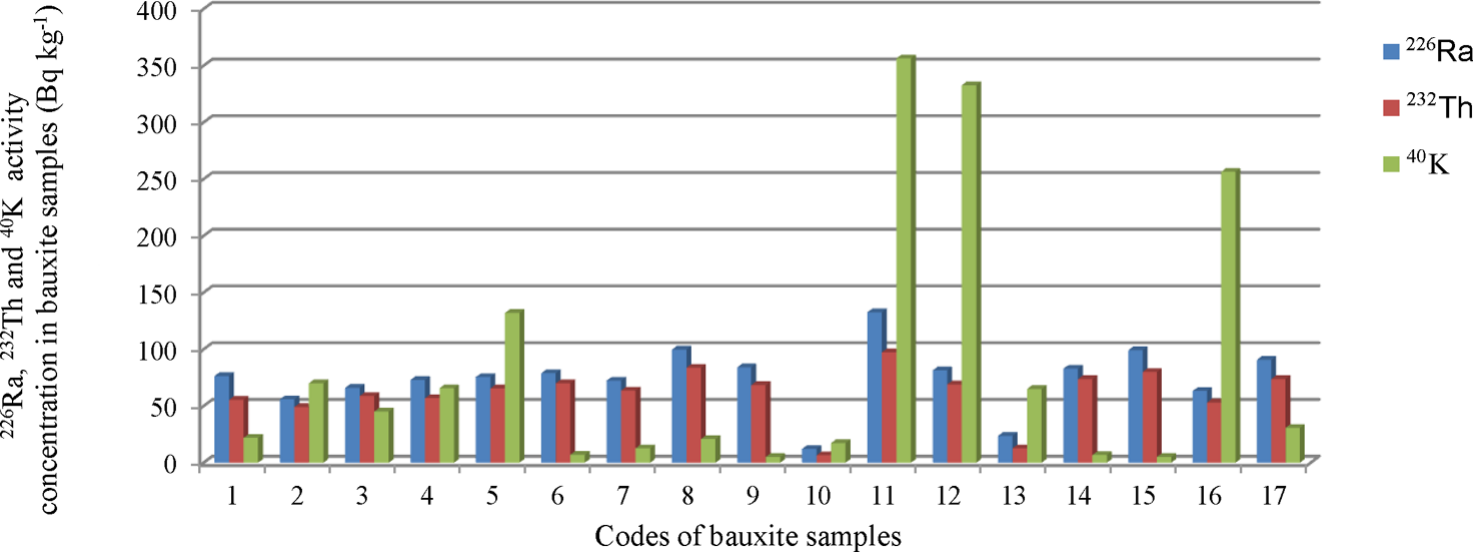

In this study, which consists of two parts, the radiological and chemical content of bauxite ore was investigated. Gamma-ray spectrometry was used in the first part of the study to determine the activity concentrations of natural radionuclides in samples taken from bauxite deposits in Turkey. The measured activity concentrations of ^226^Ra, ^232^Th, and ^40^K varied from 12.1 ± 2.5 to 132.6 ± 11.4 Bq kg^–1^ with a mean of 78.4 ± 8.3 Bq kg^–1^, 6.5 ± 0.5 to 97.3 ± 9.9 Bq kg^–1^ with a mean of 64.5 ± 5.9 Bq kg^–1^, and 5.1 ± 0.0 to 356.4 ± 40.1 Bq kg^–1^ with a mean of 52.6 ± 5 Bq kg^–1^, respectively. The calculated mean radium equivalent activity value is 177.4 Bq kg^–1^, the mean gamma dose rate absorbed in the air is 78.8 nGy h^–1^, the mean corresponding annual effective dose value is 96.6 μSv y^–1^, and the mean lifetime risk of cancer is 3.85 × 10^–4^. The chemical content of the bauxite samples was determined in the second part of the study using X-ray fluorescence spectrometry (XRF). For which sectors in the industry the existing bauxite deposits in Turkey can be used was investigated based on this information.

## 1 Introduction

Depending on their location and quality of living, people are continually exposed to cosmic and terrestrial radiation throughout their lifetimes as a result of radioactive irradiation. Most of the radiation exposure is gamma radiation emitted by radionuclides in the natural ^238^U, ^232^Th, ^235^U series, and radioactive ^40^K, which have been present since the beginning of the world. Knowing the natural radionuclide concentrations of crust-origin bauxite, which has an important place in world trade, and using such ores as raw materials will contribute to the development of relevant standards. The production of metals needed by the extraction and processing of minerals, which are important activities affecting human health, also has a large share in the country’s economy. Ores originating from the crust can adversely affect the environment they are in, depending on the concentration of natural radionuclides and the chemical components they contain. Bauxite ore is processed by extracting it from mineral deposits in the open field. Of the world’s bauxite reserves, 90% of which are used to obtain aluminum, the rest is used in the chemical, refractory, abrasive, and cement industries. Bauxite ore used in the production of aluminum, which is considered to be of exploitable value, generally contains 30–60% aluminum.^1^ Aluminum, which is found in the form of different minerals in nature, is a soft and light metal that is the most widely used after iron in the industry and has a wide area of use due to its high electrical and thermal conductivity, low density, ability to turn into thin plates, and resistance to corrosion.^2^ Especially because of its light weight, aluminum has a wide range of use in the transportation and construction sectors, which require high-strength properties.^3−6^ Bauxite, which is the main ore used in the production of aluminum metal, has become an indispensable raw material for many sectors. The corrosion of iron used as a building material seems to increase the use of aluminum day by day. The activity concentrations of ^226^Ra, ^232^Th, and ^40^K radioisotopes naturally found in bauxite ore vary from region to region and according to the geological structure of the soil.^7^ It is very important to determine the activity levels of natural radionuclides in the ore to evaluate the possible risks to human health in the long term. Although there are studies in the literature to determine the natural radioactivity levels and chemical contents of bauxite ore, no detailed study has been found to determine the natural radioactivity levels of all bauxite deposits in Turkey.^8−21^ This study aims to establish a radiological database in bauxite ores in Turkey and investigate the economic value of the chemical content of existing bauxite.

## 2 Materials and Methods

### 2.1 Study Area and Sampling

South America, Australia, the Caribbean, Guyana, Brazil, Guinea, and Jamaica in West Africa are the leading countries in terms of bauxite reserves. Other than the abovementioned, China, India, Brazil, Russia, and Venezuela are among the countries with bauxite reserves.^22^ In a report published in 2018 by the General Directorate of Mineral Research and Exploration of Turkey, it was stated that the probable bauxite reserve in Turkey is 422 million tons, about 15% of which is workable.^23^ Bauxite deposits in Turkey are open pit. The depth at which bauxite ore is extracted varies from the surface to a depth of 150 meters depending on the thickness of the vein. While digging and shoveling is sufficient near the surface, as it gets deeper, bauxite is extracted by blasting dynamite and digging tunnels at deeper levels.

**Figure 1:**
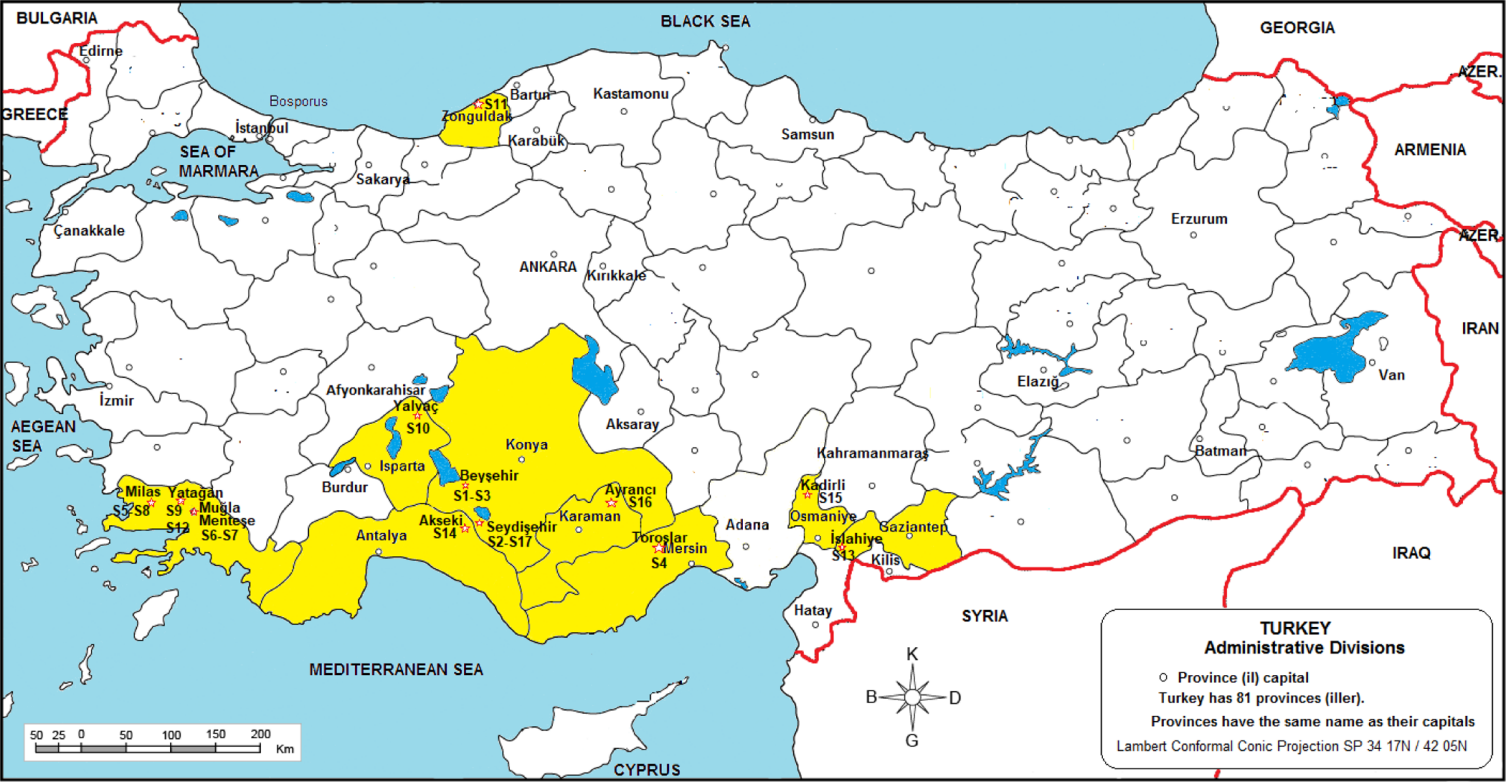
Map showing the codes of the ore beds from which the bauxite samples were taken for spectrometric measurements.

The mineral deposits from which the bauxite samples were taken and actively operated in Turkey were coded as (Konya–Seydişehir I) S-1, (Konya–Seydişehir II) S-2, (Konya–Beyşehir) S-3, (Mersin–Toroslar) S-4, (Muǧla–Milas) S-5, (Muǧla–Menteşe I) S-6, (Muǧla–Menteşe II) S-7, (Muǧla–Milas II) S-8 , (Muǧla–Yataǧan I) S-9, (Isparta–Yalvaç) S-10, (Zonguldak–Merkez (Center)) S-11, (Muǧla–Yataǧan II) S-12, (Gaziantep–İslahiye) S-13, (Antalya–Akseki) S-14,(Adana–Osmaniye–Kadirli) S-15, (Karaman–Ayrancı) S-16, and (Konya–Seydişehir) S-17, as shown in Figure 1. The bauxite samples obtained from the mine site were crushed with the help of a crusher and turned into homogeneous powder. The samples were placed in tared 6 × 5 standard sample containers and then labeled. Before starting the gamma-ray spectrometric measurements, the sealed samples were stored for 1 month to reach radioactive equilibrium of ^226^Ra, ^232^Th, and their decay products.

### 2.2 Spectrometric Measurements

In this study, the GEM30P model HPGe gamma spectrometer system belonging to the ORTEC company, which we have also measured in other studies, was used. The energy calibration of the γ-ray spectrometer was carried out by using point sources (^60^Co, ^137^Cs, and ^241^Am). Full-energy peak (FEP) efficiency calibration of the γ-ray spectrometer was performed using reference materials RGU-1 (U-ore), RGTh-1 (Th-ore), and RGK-1 (K2 SO4) supplied by the International Atomic Energy Agency. Each of these reference materials was placed in an identical polyethylene bottle of 118 cm^3^ volume and counted for 10,000 s to obtain a good counting statistic, which means that uncertainties in the counting rates of interested γ-ray peaks were less than 3% at the 95% confidence level.^24^ This HPGe detector has 30% relative efficiency. The energy resolution is 0.85% at 122 keV and 1.85% at 1.33 MeV. The detector is cooled using a 30 L liquid nitrogen container. It is useful in the energy range of 40 keV to 10 MeV. A protective coating with 9.5 mm steel and lead armor with a thickness of 101 mm was used to prevent the gamma rays of the natural background arising from cosmic rays and the environment from reaching the detector. The inner surface of the lead armor was coated with 0.5 mm of tin and a 1.5 mm copper plate to prevent the X-rays generated by gamma rays interacting with the lead from reaching the detector.

### 2.3 Radiological Parameters

The equivalent radium activity (Ra_eq_ in terms of Bq kg^–1^) value used to compare radioactivity from radium, thorium, and potassium from the uranium decay series was calculated using eq 1.^25^
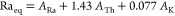
1

Wherein *A*_Ra_, *A*_Th_, and *A*_K_ are the specific activities of ^226^Ra, ^232^Th, and ^40^K, respectively. When defining equivalent radium activity, it is assumed that 10 Bq kg^–1^ of ^226^Ra, 7 Bq kg^–1^ of ^232^Th, and 130 Bq kg^–1^ of ^40^K generate equal amounts of equal gamma-ray dose rates.^26^

The gamma dose rate (*D* in terms of nGy h^–1^) absorbed from the air at 1 m above the surface originating from ^238^U, ^232^Th, and ^40^K in the crust and its derivatives and the corresponding annual effective dose (AED in terms of μSv y^–1^) value was calculated using data and eqs 2 and 3 from UNSCEAR 2008 and European Commission 1999 reports.^27,28^
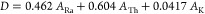
2

Considering that worldwide, people spend 20% of their lives outdoors, the conversion for the absorbed gamma dose rate was calculated using eq 3, taking into account 0.7 (Sv/Gy) and the outdoor exposure factor (0.2).^29^
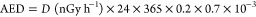
3

To predict the probability of cancer, which is a disease of our age, caused by exposure to radioactivity, the Lifetime Cancer Risk (LCR), i.e., cancer risk for any person, was calculated using eq 4 based on the AED.
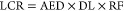
4wherein DL is the average human lifespan of 78 years.

RF is a risk factor given as 0.057 Sv^–1^ for stochastic effects that produce low background radiation.^30^

### 2.4 Chemical Measurements

Chemical measurements were performed on a high-quality Zetium XRF spectrometer (Spectro Xepos) in the Eczacıbaşı Esan chemical laboratory.

## 3 Results and Discussion

### 3.1 Activity Concentrations of ^226^Ra, ^232^Th, and ^40^K

It is important to choose the clean peak of the radionuclide to be analyzed in the gamma spectrometer. A clean analytical peak means that a peak that does not interfere with the gamma contribution of radionuclides belonging to the uranium–radium decay chain or other natural radioactive series that may exist in the samples to be analyzed and has a high probability of emitting gamma. In the calculation of the radioactivity of these radionuclides, the photopics formed by the decay products of the ^238^U and ^232^Th series in the spectrum are used. After the radioactive equilibrium between ^238^U and ^226^ Ra, the activity of the main nucleus ^238^U can be equivalent to the activity concentrations of ^226^Ra and other distant products. In order to calculate the activity concentration of ^226^Ra from its self-published gamma photopic with 186.2 keV energy, the contribution of the interfering ^235^U from the photopic with 185.7 keV energy should be subtracted. The photopic of ^226^Ra with an energy of 186.2 keV was used to calculate the activity concentration of ^238^U.

In this study, the activity concentration of ^232^Th was calculated by taking the arithmetic average of the 911.2 keV energy photopic of ^228^Ac and the 583.2 keV energy photopic of ^208^Tl. In the calculation of the activity concentration of ^40^K, the 1460 keV gamma energy photopic specific to this radionuclide was used. The activity of the respective radionuclides was calculated using eq 1.^31^
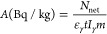
5

Here, *A* in the formula is the calculated activity, *N*_net_ is the net peak area, ε_γ_ is the detector efficiency for the peak of interest, *t* is the count time, *m* is the mass of the sample (kg), and *I*_γ_ is the probability of emitting gamma energy.

The activity values obtained are shown in Table 1. As can be seen from Table 1, mean activity concentrations in bauxite samples are 78.4 ± 8.3 Bq kg^–1^ for ^226^Ra; the smallest value was determined as 12.1 ± 2.5 Bq kg^–1^ in the (Isparta–Yalvaç) S-10 coded sample, and the highest value was 132.6 ± 11.4 Bq kg^–1^ in the (Zonguldak–Merkez (Center)) S-11 coded sample. The mean activity concentration for ^232^Th is 64.5 ± 5.9 Bq kg^–1^; the smallest value was determined as 6.5 ± 05.1 Bq kg^–1^ in the (Isparta–Yalvaç) S-10 coded sample, and the highest value was 97.3 ± 9.9 Bq kg^–1^ in the (Zonguldak–Merkez (Center)) S-11 coded sample. The activity concentration for ^40^K was determined to vary between 2.7 ± 0.3 and 356.4 ± 40.1 Bq kg^–1^, and the highest measured value was found in the (Zonguldak–Center) S-11 coded sample. It is noteworthy that the highest activity value of the ^226^Ra, ^232^Th, and ^40^K radionuclides is at the station coded S-11. Moreover, it is known that many ores other than bauxite are mined in the region where this station is located, and the reason is thought to be related to the geological structure of the place. In the Environmental Report published in 2000 (Environmental Protection Agency), it was reported that the activity concentration of ^238^U in bauxite ore varies between 162.8 and 273.8 Bq kg^–1^.^32^ In the reports published by the International Atomic Energy Agency in 2003, as for that, it was reported that the activity concentrations of uranium and thorium series radionuclides in bauxite ore vary at 10–900 and 35–1400 Bq kg^–1^, respectively, while the activity concentration of ^40^K radionuclide varies between 10 and 600 Bq kg^–1^.^33^ It was discovered that the typical activity concentrations discovered in this investigation fell within the range of numbers reported in the reports and taken as a standard. Remarkably, the mean activity concentration for ^238^U in the bauxite samples used in the experiment is approximately 2.5 times the world average, and the mean activity concentration for ^232^Th is approximately 2 times the world average. The mean activity concentration of the ^40^K radioisotope, which is well below the world average, is 52.6 ± 5 Bq kg^–1^. Similar study results found in the literature are shown in Table 2.

**Table 1:** ^226^Ra, ^232^Th, and ^40^K Activity Concentrations Measured in the Bauxite Samples

	radioactivity values measured in bauxite samples (Bq kg^–1^)		
sample code	^226^Ra	^232^Th	^40^K
S-1	76.4 ± 8.1	55.4 ± 4.8	21.9 ± 1.9
S-2	55.8 ± 5.1	49.1 ± 4.0	70 ± 7.1
S-3	66 ± 5.9	58.8 ± 5.0	45.1 ± 3.9
S-4	73 ± 8.0	56.9 ± 5.1	65.7 ± 6.8
S-5	75.5 ± 8.1	65.5 ± 7.2	132 ± 11.1
S-6	78.9 ± 8.8	70 ± 8.1	7 ± 0.2
S-7	72.3 ± 7.9	63.6 ± 5.9	12.7 ± 0.9
S-8	99.7 ± 9.6	83.7 ± 9.1	20.8 ± 2.5
S-9	84.1 ± 9.2	68.4 ± 6.2	5.1 ± 0.0
S-10	12.1 ± 2.5	6.5 ± 0.5	17.3 ± 1.8
S-11	132.6 ± 11.4	97.3 ± 9.9	356.4 ± 40.1
S-12	81.37 ± 7.2	69 ± 5.8	332.7 ± 39.7
S-13	23.7 ± 3.9	12.7 ± 1.7	65 ± 5.8
S-14	82.87 ± 9.1	73.7 ± 7.1	6,8 ± 0.5
S-15	99.2 ± 10.0	80 ± 9.4	5,1 ± 0.4
S-16	63.21 ± 7.1	53.1 ± 6.1	256.5 ± 21.9
S-17	90.73 ± 8.6	73.7 ± 7.1	30.7 ± 3.1
min	12.1 ± 2.5	6.5 ± 0.5	5.1 ± 0.0
max	132.6 ± 11.4	97.3 ± 9.9	356.4 ± 40.1
average	78.4 ± 8.3	64.5 ± 5.9	52.6 ± 5.0

**Table 2:** Comparison of the Obtained Data with Similar Studies in the Literature

	activity concentrations in bauxite samples (Bq kg^–1^)			
location	^226^Ra	^232^Th	^40^K	references
Hungary	419	256	47	(8)
Greece	230	387	17	(9)
Guinea and India	62	328		(10)
China	370	400	64	(11)
Brazil	64	154	9.4	(12)
Turkey–Seydişehir	89	227	106	(13)
Saudi Arabia	102. 2	156.3	116.8	(14)
Western Australia	120–350	450–1050	30–70	(15)
Turkey–Kas	164.8	125.8	53.7	(17)
Qena (Egypt)	12–28	13–53	950–980	(20)
Jamaica	10–900	35–1400	10–600	(21)
Guyana	55 + 2	250 + 10	27 + 1	(29)
this study	78.4 ± 8.35	64.5 ± 5.95	52.6 ± 5	

**Figure 2:**
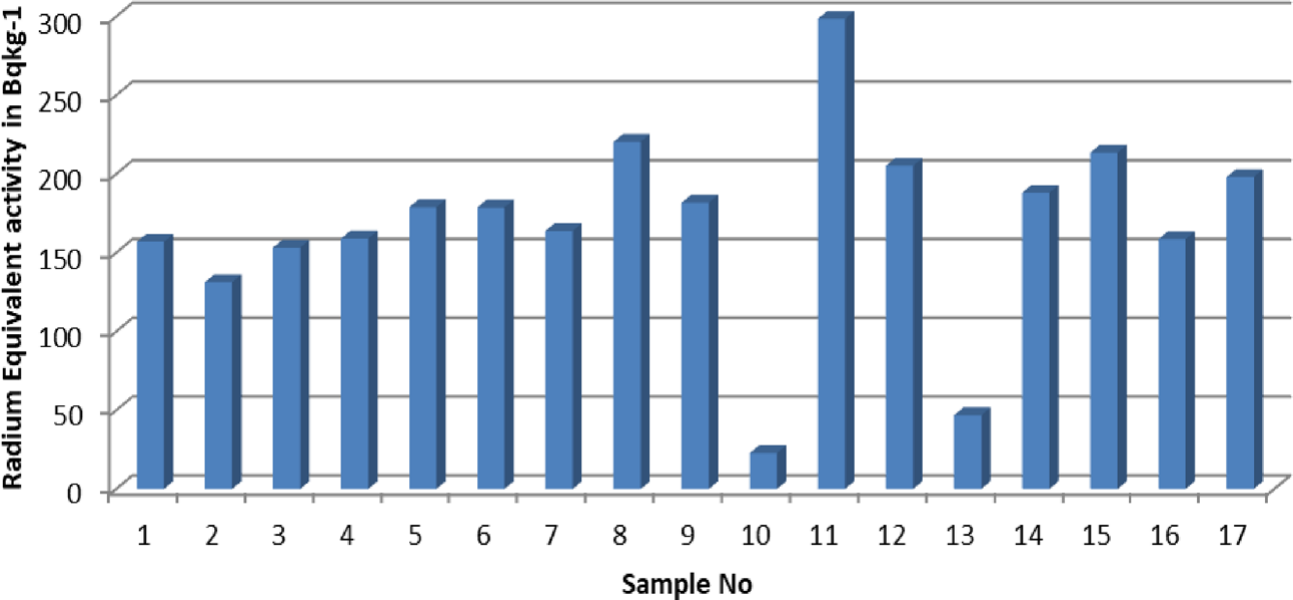
Equivalent radium activity values calculated in the bauxite samples.

The calculated equivalent radium activity value ranges from 22.8 to 220.4 Bq kg^–1^ with a mean of 177.45 Bq kg^–1^. The calculated mean value is considerably smaller than the reference value of 370 Bq kg^–1^. In the calculations based on activity values in bauxite samples, the mean absorbed gamma dose rate and the annual effective dose was found to be 78.85 nGy h^–1^ and 96.69 μSv y^–1^, respectively. The bar graph of the calculated equivalent radium activity values is shown in Figure 2.

**Figure 3:**
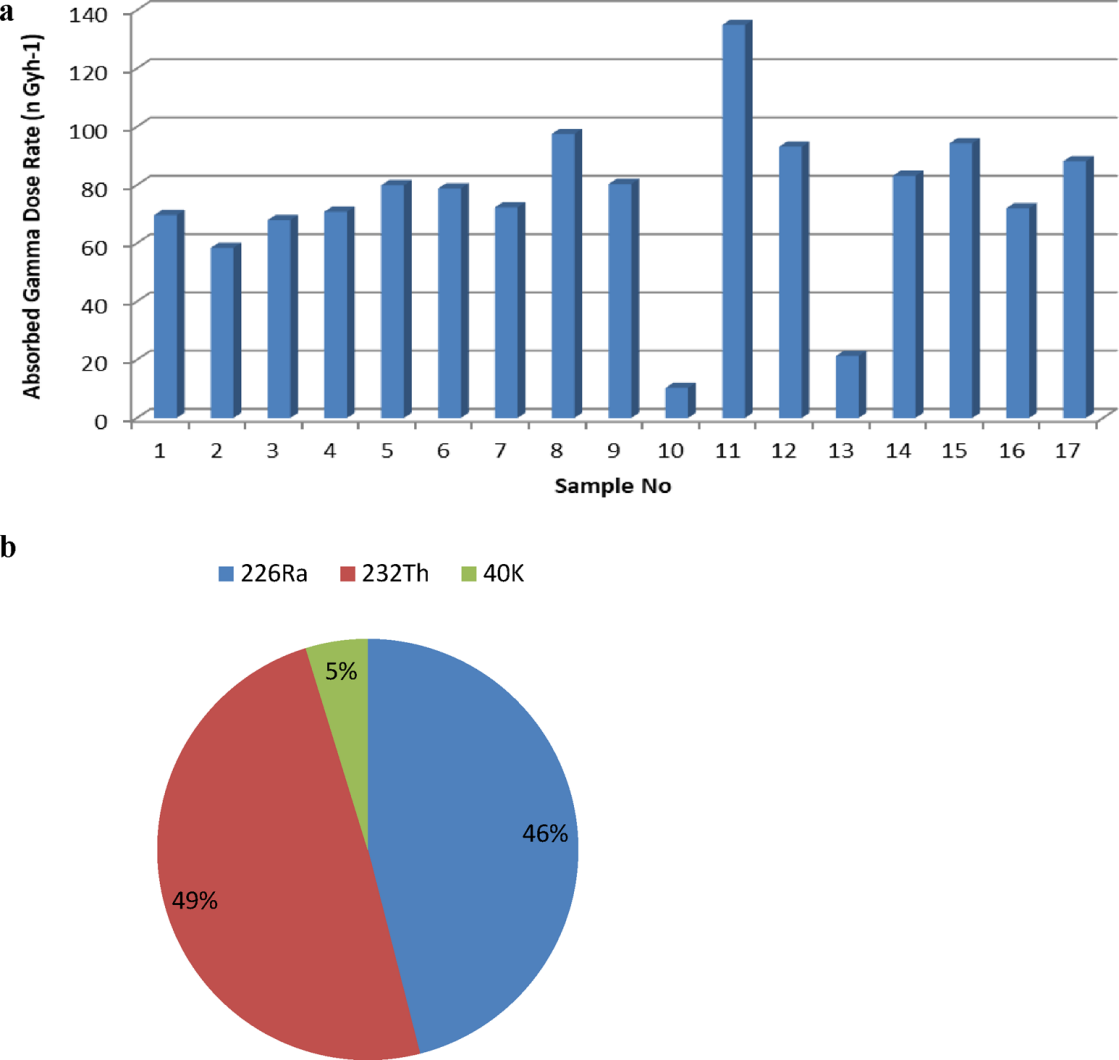
(a) Absorbed gamma dose rate values calculated in the bauxite samples. (b) Gamma dose rate rates from ^226^Ra, ^232^Th, and ^40^K in the bauxite samples.

In the UNSCEAR 2008 report, the mean absorbed gamma dose rate (*D*) in air 1 m above the surface originating from the radioisotopes ^238^U, ^232^Th, and ^40^K in the soil was announced as 60 nGy h^–1^. The bar graph of the calculated absorbed gamma dose rate values is shown in Figure 3a,b. As seen in Figure 3b, the largest contribution to the absorbed dose rate is from ^232^Th.

**Figure 4:**
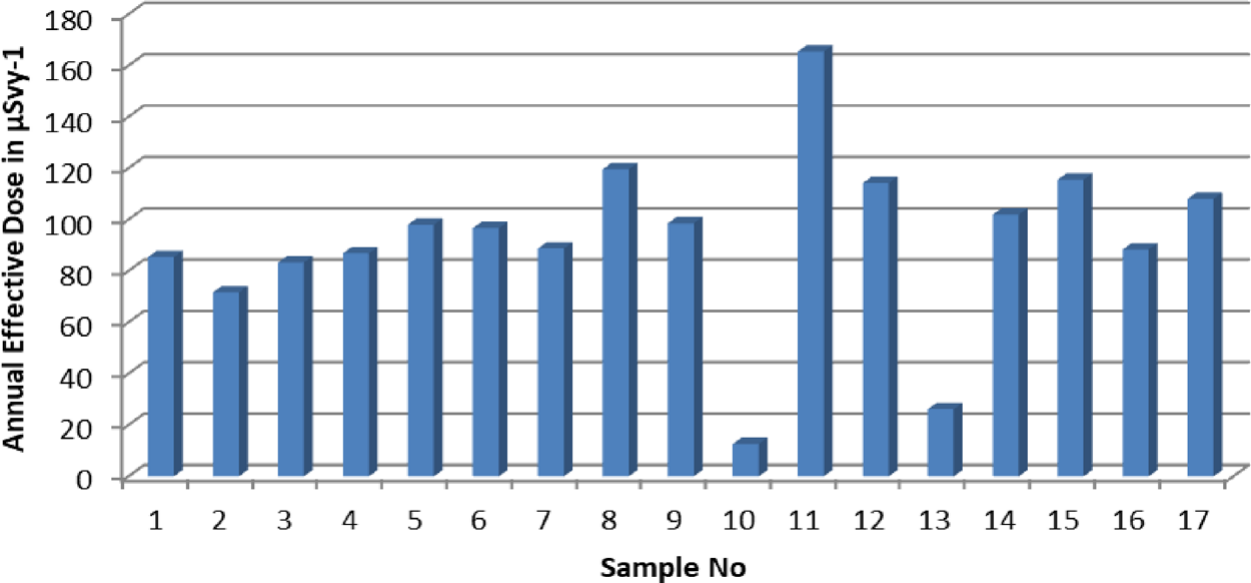
Annual effective dose values calculated in the bauxite samples.

**Figure 5:**
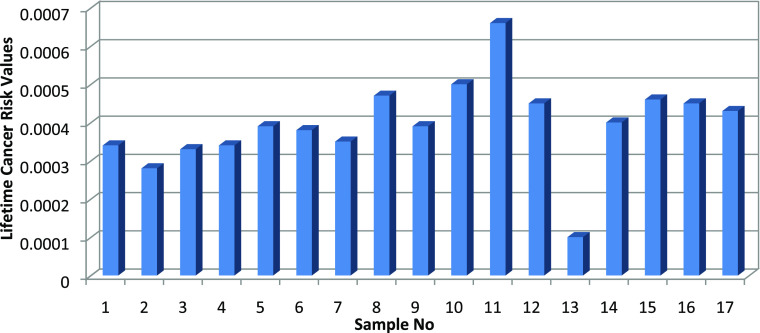
Lifetime cancer risk values.

According to same report, the annual effective dose rate (AED) of 70 μSv y^–1^ and the risk of permanent cancer of 2.9 × 10^–4^ was announced. In the evaluation made according to this report, it is seen that the calculated radiation dose values are slightly higher than the values accepted as criteria. The column graph of the calculated annual effective dose values is shown in Figure 4. Values obtained for lifetime cancer risk (LCR) ranged from 6.6 × 10^–4^ to 0.5 × 10^–4^, with a mean of 3.8 × 10^–4^. This calculated value is slightly higher than the world average accepted reference value. The bar graph of the calculated lifetime cancer risk values is shown in Figure 5.

### 3.2 Chemical Analysis

As shown in Table 3, the average percentages of SiO_2_, Al_2_O_3_, Fe_2_O_3_, TiO_2_, CaO, MgO, Na_2_O, and K_2_O compounds in bauxite samples were found as 9.87, 52.80, 22.70, 3.06, 0.50, 0.33, 0.17, and 0.39, respectively. In addition to being used in different fields depending on its chemical composition in the industry, a large part of it is used in the production of aluminum metal. The production of aluminum metal depends on the production of Al_2_O_3_, and ore containing 50–60% Al_2_O_3_ is processed. There are also small amounts of CaO, MgO, Na_2_O, and K_2_O compounds with SiO_2_, Fe_2_O_3_, and TiO_2_ in the ore.^34^ The quality of bauxite has been described according to many criteria. However, the most widely used one is the classification according to silica module (% Al_2_O_3_/% SiO_2_) and %Fe_2_O_3_ grade. Accordingly, if % Al_2_O_3_/% SiO_2_ > 20, it is defined as a high-alumina ore; if % Al_2_O_3_/% SiO_2_ = 10–20, it is defined as an aluminum ore; if %Al_2_O_3_/% SiO_2_ = 4–10, it is defined as a siliceous (industrial) ore; if % Al_2_O_3_/% SiO_2_ < 4, it is defined as a high-silica ore; if % Fe_2_O_3_ > 25, it is defined as a high-iron ore; if % Fe_2_O_3_ = 10–25, it is defined as a ferrous ore; and if % Fe_2_O_3_ < 10, it is defined as a low-iron ore. If the ratio of %Al_2_O_3_/% SiO_2_ in bauxite is greater than 7, it means that the ore is more economical.^35^ According to Table 4, which shows the chemical composition of bauxite according to its usage areas, it can be predicted which sectors the bauxite mine extracted in Turkey is useful.^36^ It is expected that the Al_2_O_3_ component of bauxite, which is used in aluminum production in the metallurgical sector, is between 50 and 55%, and the SiO_2_ component is at most 15%; in this case, the chemical composition of the samples coded S-5, S-8, S-14, S-15, S-16, and S-17 shows that the bauxite mine extracted in these regions can be used in the production of aluminum in the metallurgical sector. It can be predicted that the samples coded S-10, S-11, S-13, and S-16, where the SiO_2_ component is above 10%, can be used in cement production. It can also easily be said that the samples coded S-4, S-8, S-14, S-15, and S-17, in which the ratio of the %Al_2_O_3_ value to the % SiO_2_ value in bauxite is greater than 7, is commercially valuable.

**Table 3:** XRF Results of the Bauxite Samples

sample code	SiO_2_ %	Al_2_O_3_ %	Fe_2_O_3_ %	TiO_2_ %	CaO %	MgO %	Na_2_O %	K_2_O %
S-4	2.96	45.91	37.16	2.76	0.09	0.23	0.17	0.24
S-5	8.58	53.74	23.16	2.55	1.29	0.17	0.47	0.70
S-8	5.59	62.51	15.85	3.69	0.08	0.31	0.08	0.03
S-10	27.38	40.39	18.16	3.14	0.22	0.23	0.08	0.12
S-11	16.44	47.24	14.76	1.64	2.63	1.40	0.11	1.30
S-13	15.70	45.45	21.25	6.55	0.09	0.21	0.10	0.13
S-14	2.31	59.09	23.98	2.43	0.12	0.17	0.10	0.05
S-15	5.87	50.76	29.05	2.57	0.19	0.26	0.08	0.06
S-16	11.42	51.00	23.40	2.41	0.16	0.21	0.45	1.10
S-17	2.42	61.89	20.26	2.84	0.10	0.12	0.05	0.18
average	9.87	52.80	22.70	3.06	0.50	0.33	0.17	0.39

**Table 4:** Chemical Composition of Bauxite According to Usage Areas

İçerik	metallurgy (%)	chemical (%)	cement (%)	refractory (%)	caustic (%)
Al_2_O_3_	50–55	min 55	45–55	84.5	80–88
Si0_2_	0.15	5–18	max 6	7.5	4–8
Fe_2_O_3_	5–30	max 2	20–30	2.5	2–5
TiO_2_	0–6	3	3	3	2–5

## 4 Conclusions

There are just a few countries in the world where bauxite ore is extracted. Utilizing the reserve resources in these countries is very important for the country and the world economy. Depending on its chemical composition, bauxite ore is utilized as a construction material in a variety of industrial sectors, particularly in the manufacturing of aluminum.

Since bauxite is from the Earth’s crust, the determination of natural radioactivity levels will contribute to the establishment of relevant standards. In this study, natural radioactivity levels of bauxite ore mining in Turkey were measured using a gamma spectrometry system. As seen in Table 2, the mean of the measurement results for activity are 78.4 ± 8.3, 64.5 ± 5.9, and 52.6 ± 5 Bq kg^–1^ for ^226^Ra, ^232^Th, and ^40^K, respectively. According to the results obtained, ^226^Ra and ^232^Th radioactivity concentrations are approximately 2–2.5 times the world average. It was observed that the lifetime cancer risk calculated based on the measured activity values of the samples is 1.3 times the world average. All in all, the naturally radioactive ^226^Ra and ^232^Th contained in bauxite ore slightly increase the activity level of the environment. The quality of commercially important bauxite ore is evaluated according to its chemical content. Chemical analysis of bauxite samples selected for the study was determined by X-ray fluorescence spectrometry (XRF). In bauxite samples, the SiO_2_ percentage is 9.87% on average, the Al_2_O_3_ percentage is 52.80% on average, the Fe_2_O_3_ percentage is 22.70% on average, the TiO_2_ percentage is 3.06% on average, the CaO percentage is 0.50% on average, the MgO percentage is 0.33% on average, the Na_2_O percentage is 0.17% on average, and the K_2_O percentage is 0.39% on average. The results of the chemical analysis prove that the bauxite ore in Turkey is commercially valuable and will contribute to aluminum production at an international level.
